# Priming and positioning of lateral roots in Arabidopsis. An approach for an integrating concept

**DOI:** 10.1093/jxb/erv541

**Published:** 2015-12-27

**Authors:** Stefan Kircher, Peter Schopfer

**Affiliations:** Department of Molecular Plant Physiology, Faculty of Biology, Albert-Ludwigs-University, Schänzlestr. 1, D-79104 Freiburg, Germany

**Keywords:** Arabidopsis, bending hypothesis, lateral root initiation, oscillation hypothesis, pattern formation, waving growth.

## Abstract

Branching by *de novo* formation of lateral roots along the primary root of Arabidopsis seedlings follows a complex longitudinal and transverse pattern. How this pattern is generated is presently under debate. The ‘bending hypothesis’ proposes that lateral root primordia are initiated by a local accumulation of auxin at the convex side of bends resulting from deflections through obstacles, gravitropic bending, or other means. In contrast, the ‘oscillation hypothesis’ proposes the existence of an endogenous clock-type oscillator mechanism producing periodic pulses of gene expression in the root tip that determine the future sites of primordium initiation. Here we report physiological experiments dissecting periodic priming signals, pre-disposing the root to rhythmic lateral root formation, from bending-mediated signals responsible for the subsequent positioning of their initiation along the growing root. While the frequency of lateral roots can be promoted by auxin in the mature root, their positioning follows a pre-formed pattern determined by previous bending. Both types of signals turn out to be necessary, complementary components in an integrating concept of lateral root patterning.

## Highlight

Lateral root pattern formation is controlled mechanistically by separate processes: oscillating priming signals determine lateral root frequency and mechanical cues determine their spatial positioning along the primary root.

## Introduction

The development of lateral roots (LRs) on the primary root of plants is presently under intense investigation chiefly for three reasons. (i) LR development represents a case of *de novo* organ formation starting from the re-initiation of meristematic activity in cells that have already entered the route towards differentiated body cells (Parizot *et al.*, 2008). (ii) LR formation demonstrates radial and longitudinal patterns along the primary root directed by positional information (Fortin *et al.*, 1989). (iii) LR formation provides a paradigm for the emergence of all-or-none cell-fate decisions on the basis of developmental steady-state conditions (Ferrell and Xiong, 2001). These phenomena concern central, insufficiently explored issues of developmental biology that are subject to ongoing debate (Van Norman *et al.*, 2013; Laskowski, 2013). It has been known since the pioneering work of Noll (1900) that LRs of dicotyledonous plants originate from a subset of pericycle cells next to the xylem pole of the stele. The roots of plants such as *Arabidopsis thaliana* (L.) Heynh. are characterized by a bilaterally symmetric (diarch) organization of the vascular tissues, i.e. two xylem poles at opposite sides of the stele and thus two opposite positions of xylem-adjoining pericycle cell files. These cell files have the potential for LR founder cell initiation by asymmetric cell division starting the generation of a LR primordium (LRP). Confined by this endogenously defined pre-pattern of rhizogenic pericycle cell files, the induction and positioning of founder cell initiation along the growing root can be controlled by endogenous and environmental factors such as hormones, light, nutrient supply, and mechanical cues (Nibau *et al.*, 2008). As already shown by Noll (1900), the radial bipolar pattern of LRs is accompanied by a longitudinal pattern of LR positioning: LRs are generally located at the convex side of bends along the primary root, caused by deflections through obstacles, gravitropic bending, or manual deformation (Ditengou *et al.*, 2008; Laskowski *et al.*, 2008; Lucas *et al.*, 2008a; Richter *et al.*, 2009). Work with an Arabidopsis line expressing a reporter gene for auxin-dependent gene activation (*pDR5:GLUCURONIDASE*) has shown that founder cell initiation is accompanied by the activation of auxin response genes in the pericycle regions where LR development takes place (Ditengou *et al.*, 2008; Dubrovsky *et al.*, 2008; Laskowski *et al.*, 2008; Richter *et al.*, 2009). This event has been localized to the border between elongation zone and maturation zone (approx. 1.5mm behind the root tip in 5-d-old seedlings), i.e. in close proximity to the zone where bending normally occurs. These and related observations have led to the view that founder cell initiation is triggered by root bending and that some unknown mechanical stimulus at the convex side of bends acts as an inducing signal via a local accumulation of auxin. In this study, we refer to this concept as the ‘bending hypothesis’.

Using an alternative approach, De Smet *et al.* (2007) have shown that the longitudinal spacing of LRs can be related to periodic fluctuations of *pDR5* reporter gene expression in a root region next to the basal border of the apical meristem (approx. 1.0mm in front of the founder cell initiation site). Thus, founder cell initiation appears to be preceded by a recurrent gene-activating priming reaction that pre-disposes pericycle cells to become founder cells at regular time intervals. Significantly, this event can be first observed in the protoxylem at both xylem poles, indicating that the restriction of the signal to one pole has not yet occurred (De Smet *et al.*, 2007). Moreno-Risueno *et al.* (2010) have shown that the putative priming signals detectable with the *pDR5:LUCIFERASE* (*pDR5:LUC*) reporter gene in a root zone comprising the basal meristem and the elongation zone (termed the ‘oscillation zone’) are succeeded by stable spots of reporter activity in the distal maturation zone, i.e. in a root segment where LRP formation later occurs (termed ‘prebranch sites’). The amplitude of these pulses depends on auxin from the root cap and affects the realization of prebranch site formation (Xuan *et al.*, 2015). Based on these results and related gene expression studies, the ‘oscillation hypothesis’ proposes that periodic pulses of a gene-activating priming reaction determine the longitudinal spacing of LRPs (Moreno-Risueno *et al.*, 2010). This poses the question of the role of root bending in LR formation. Evidently, the oscillation hypothesis cannot explain the correlation between bending and the unilateral positioning of LRs at the sites of bending. Conversely, the bending hypothesis cannot explain the correlation between recurrent *pDR5* activity pulses in the oscillation zone and subsequent LRP initiation in the distal maturation zone. Remarkably, this discrepancy concerns the fundamental question of whether LR formation depends on an endogenous oscillating time-keeping mechanism in addition to environmental cues. Here, we present physiological experiments designed to clarify this problem.

## Materials and methods

### Growth conditions

After a stratification treatment (2 d at 5°C), sterilized (75% ethanol) seeds of *Arabidopsis thaliana* (Columbia-0) were germinated and the seedlings grown at 25°C in continuous light (100 µmol m^−2^ s^−1^) from fluorescent white light tubes arranged sideways in the growth chamber (Sanyo) on vertical agar (10g l^−1^) plates with half-strength Murashige and Skoog medium (including vitamins; obtained from Duchefa Biochemie, The Netherlands, cat. no. M0222), 10mM MES buffer (pH 6.1) and changes as indicated. Root growth within the agar medium instead of at the surface was achieved by keeping the plates in the horizontal position during the first 2 d after initiation of germination allowing the root tip to grow into the agar. A clinostat driven by a step motor was used to rotate plates continuously in the vertical position. A similar ‘reeling device’ was used for periodically rotating plates back and forth by 90° for adjustable time intervals between instant rotations. Prenoxylbenzoic acid was obtained from W. Michalke (Michalke *et al.*, 1992).

### Measurement of primordium number and root elongation

For counting primordia, roots were stained with acetocarmine prepared by boiling with reflux 0.5g carmine in 100ml 45% acetic acid for 1h (Hackett and Stewart, 1969). After mechanically fixing the roots on the plate by spraying with hot 0.8% agar, chemical fixation with formalin/acetic acid/ethanol/water (5:5:45:45 by vol.), and staining for 24h followed by extensive washing with water, the plates were inspected using a microscope (see Supplementary Fig. S1 at *JXB* online). Root elongation was measured with a ruler (±0.5mm) after marking segments of interest at the backside of plates. Undulating roots (segments) were pulled straight after fixation.

### Measurement of luciferase activity

Transgenic seeds expressing the *pDR5:LUC* reporter gene used in the experiments of Fig. 5C, Supplementary Figs S2 and S3 were obtained from the Benfey lab (Moreno-Risoeno *et al.*, 2010). Luciferase activity in living seedlings was visualized by spraying seedlings with 2mM luciferin (Biosynth AG, Switzerland) solution containing 0.01% Triton X-100. Videos showing the development of prebranch sites were obtained with a VersArray XP camera system (Roper Scientific, USA) operated at exposure times of 5min (binning 2) interrupted by periods of 14min light (50 µmol m^−2^ s^−1^) and 1min darkness. Image data were processed using ImageJ (NIH, USA).

### Statistics

Data points represent means ± standard error of four to six independent experiments with samples comprising 10 seedlings. If not indicated, standard errors were typically between 5 and 12%.

## Results

**Fig. 1.**
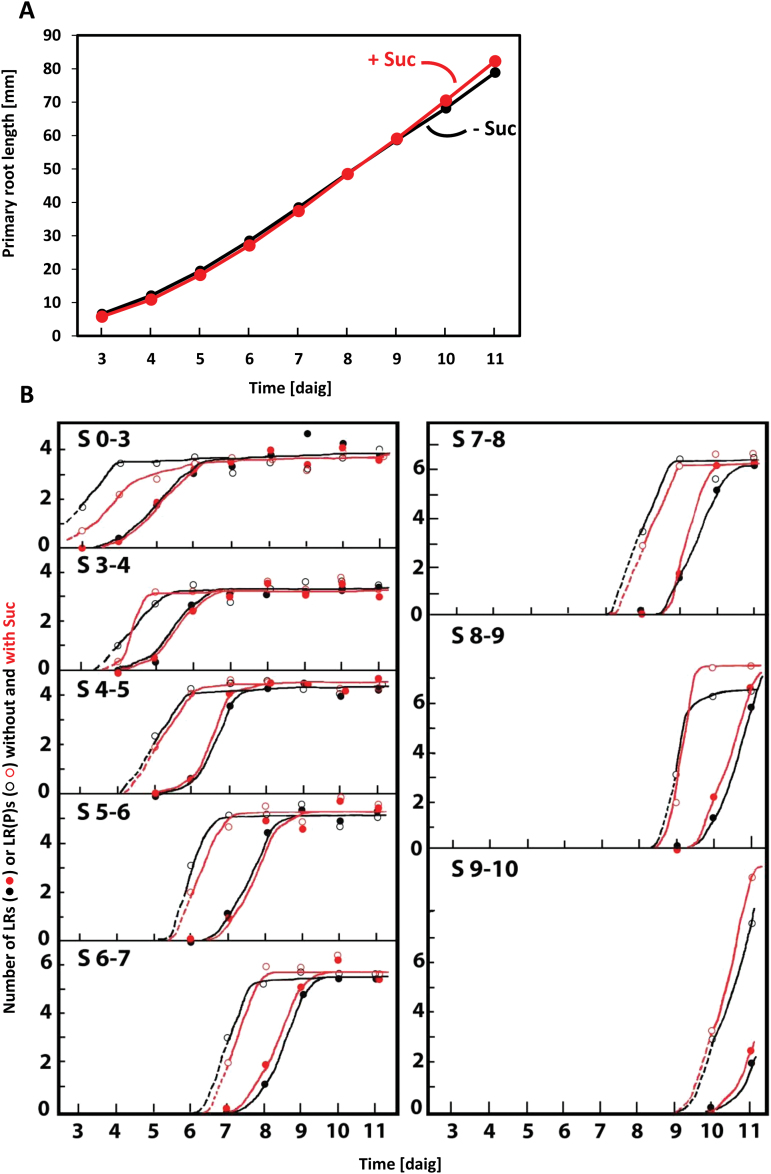
Primary root elongation (A) and temporal pattern of LR formation (B) of Arabidopsis seedlings growing on agar plates without or with 10g l^−1^ sucrose (Suc). The appearance of LRPs (open symbols) and emerged LRs (closed symbols) was followed in the primary root segments (approx. 11mm long) produced during1d intervals. Thus, S 0–3 refers to the root segment produced during the first 3 daig, S 3–4 to the root segment produced during the 4 daig, etc. Standard errors were in the range of data points (A) or in the range of 5–12% (B).

In Arabidopsis seedlings the initiation of LR development up to incipient primordia is normally restricted to a narrow zone of 1–7mm behind the root apex, and their progression into emerged LRs follows an acropetal spatial gradient (Dubrovsky *et al.*, 2006). However, the temporal pattern of LR development illustrated in Supplementary Video S1 and quantitatively documented in Fig. 1 has not yet been reported in detail. Under the experimental conditions used here, the primary root grows with an accelerating rate up to 5 d after initiation of germination (daig), followed by a constant rate of 10.8±0.5mm d^−1^ in the period of 5–10 daig (Fig. 1A). In order to include early stages of LRPs in the analysis, acetocarmine staining of meristematic tissues (Hackett and Steward, 1969) was used for detecting LRPs from stage II onwards (classification according to Malamy and Benfey, 1997; Supplementary Fig. S1). Moreno-Risueno *et al.* (2010) have shown that, although prebranch site formation determines the positions of LRPs, not all prebranch sites might subsequently produce LRs. When the pattern of prebranch site formation detectable by *pDR5:LUC* imaging (Moreno-Risueno *et al.*, 2010) were compared with the pattern of LRPs detectable by acetocarmine staining 1 d later, there was found to be complete correspondence between persistent spots of LUC activity and stainable LRPs. Weaker, non-persistent LUC spots (disappearing less than 24h after their generation) that were occasionally observed did not lead to LRPs (Supplementary Fig. S2). How the growing root is continuously populated with LR(P)s (LRs+LRPs) in the period of 5–10 daig is shown in Fig. 1B. New root portions (approx. 11mm long) produced consecutively in 1-d intervals are furnished with slightly increased numbers of LRPs (five to six LRPs during days 5–9 and eight to nine LRPs at day 10) and 98±2% of these LRPs develop subsequently into emerged LRs. Thus, in contrast to previous reports (Dubrovsky *et al.*, 2006; Lucas *et al.*, 2008b), we did not observe significant numbers of arrested or delayed LRPs under the standard experimental conditions used here. The obvious deviations of these results from previous reports may result from differences in the vigour of seed batches and/or growth conditions used. For instance, root growth and LR formation critically depend on the type of light source, the fluence rate, and the duration of the daily light period. At suboptimal light conditions, sucrose feeding increases the growth rate and LR formation (unpublished results). However, at sufficiently high white light fluence rates, the presence of 1% sucrose in the medium had no significant effect on primary root growth and LR formation (Fig. 1). These data demonstrate that under the conditions used here, root development in 5- to 10-d-old seedlings proceeds with a slightly increasing rate resulting in the establishment of an average LR(P) frequency of 6.5±0.8 d^−1^, corresponding to an average time interval of 3.7±0.4h, and an average LR(P) density of 0.61±0.05mm^−1^, corresponding to an average space interval of 1.6±0.2mm between successive LRPs. This provides a time frame of nearly steady LR formation allowing the investigation of the influence of experimental factors on the temporal and spatial pattern of LR generation under defined experimental conditions.

**Fig. 2.**
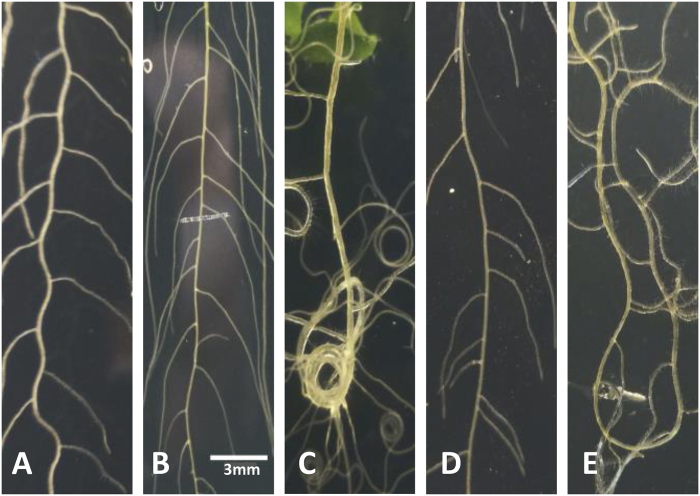
Modification of root architecture by physical manipulations affecting the relative influences of gravitropism (promoting straight growth downward) and thigmotropism (promoting slanting growth sideward). (A) Moderate friction between the root tip and an agar (10g l^−1^) surface inclined by 45° causes regular periodic alterations between lateral slanting and gravitropic re-adjustment, i.e. waving. (B) Preventing unilateral friction by forcing the root to grow vertically within an agar (10g l^−1^) medium causes straight growth. (C) Maximum friction between the root tip and a horizontal, impenetrable agar (15g l^−1^) surface causes continuous slanting, i.e. coiling. (D) Low friction between root tip and a vertical agar (10g l^−1^) surface causes shallow, irregular bending. (E) Strong friction between root tip and a hard agar (15g l^−1^) surface inclined by 45° causes chaotic meandering.

In support of the bending hypothesis, LR initiation has often been related to the waving phenomenon, i.e. regular periodic bending of the primary root growing downward on the surface of a solid medium (Okada and Shimura, 1990). Strikingly, waving growth is accompanied by a regular left–right alternating formation of LRs at the top of the convex side of bends (‘one bend–one LR’; Fig. 2A) (De Smet *et al.*, 2007; Ditengou *et al.*, 2008; Laskowski *et al.*, 2008; Richter *et al.*, 2009; Moreno-Risueno *et al.*, 2010). A similar bending pattern has been produced by growing roots under periodically alternating gravitropic stimulation (6h intervals; Lucas *et al.*, 2008a). Waving represents a special mode of oscillating root growth where gravitropic and thigmotropic (friction avoiding) changes in growth direction alternate in a delicate interaction that can be promoted by growing the seedlings on the surface of a 45° inclined agar plate (Okada and Shimura, 1990). If this situation is not met, the root demonstrates almost straight growth downward (embedded in a vertical plate; Fig. 2B), continuous curving in one direction (coiling, on the surface of a horizontal impenetrable plate; Fig. 2C) previously described as a mutant trait of genotypes deficient in gravitropism (De Smet *et al.*, 2007; Ditengou *et al.*, 2008; Okada and Shimura, 1990), irregular meandering (on the surface of a vertical plate; Fig. 2D), or chaotic curving (on the surface of a hard, inclined plate; Fig. 2E). LRs are produced similarly under all these conditions, but remarkably, never at the concave side of curves. It has been reported that the frequency of seedlings with LRs at convex positions close to the root tip can be increased by manually bending root tips from which the apical meristem was removed (Ditengou *et al.*, 2008). To test whether meristem amputation promotes bending-mediated LR formation and not just positioning, the effects of manual bending and meristem amputation were measured separately and in combination. Table 1 shows that meristem amputation increased the number of LRs in a region of 0–5mm behind the root tip similarly with and without bending while bending did not significantly increase the number of LRs but caused a convex positioning of LRs in this region in amputated and not-amputated root tips. Collectively these observations shed doubt on a simple causal relationship between root bending and the longitudinal pattern of LR formation as proposed by previous investigators. In order to clarify this point, three experiments were designed.

**Table 1.** Effect of manual root bending on LR production without and with root meristem amputation The 3–5mm tip region of the root of 5-d-old seedlings was bent (130–160^o^) using fine forceps in intact roots or after cutting the tip (0.5mm), which stops growth immediately. In intact control seedlings the position of cutting was marked on the backside of the plate. LR numbers in the whole root and the apical 5-mm zone were counted after a further 5 d when all initiated LRPs were transformed into LRs. After bending, LRs were produced on the convex side in 100% of cut und intact roots (52% in unbent roots). Shown are means from 10 independent experiments with 8–10 seedlings for each treatment.

Root region analysed	Number of lateral roots			
	Intact	Intact, bent	Cut	Cut, bent
Total above cutting	13.2±0.4	13.4±0.4	15.4±0.7	15.8±1.1
5mm above cutting	3.3±0.1	3.6±0.2	5.1±0.3	5.4±0.2

**Fig. 3.**
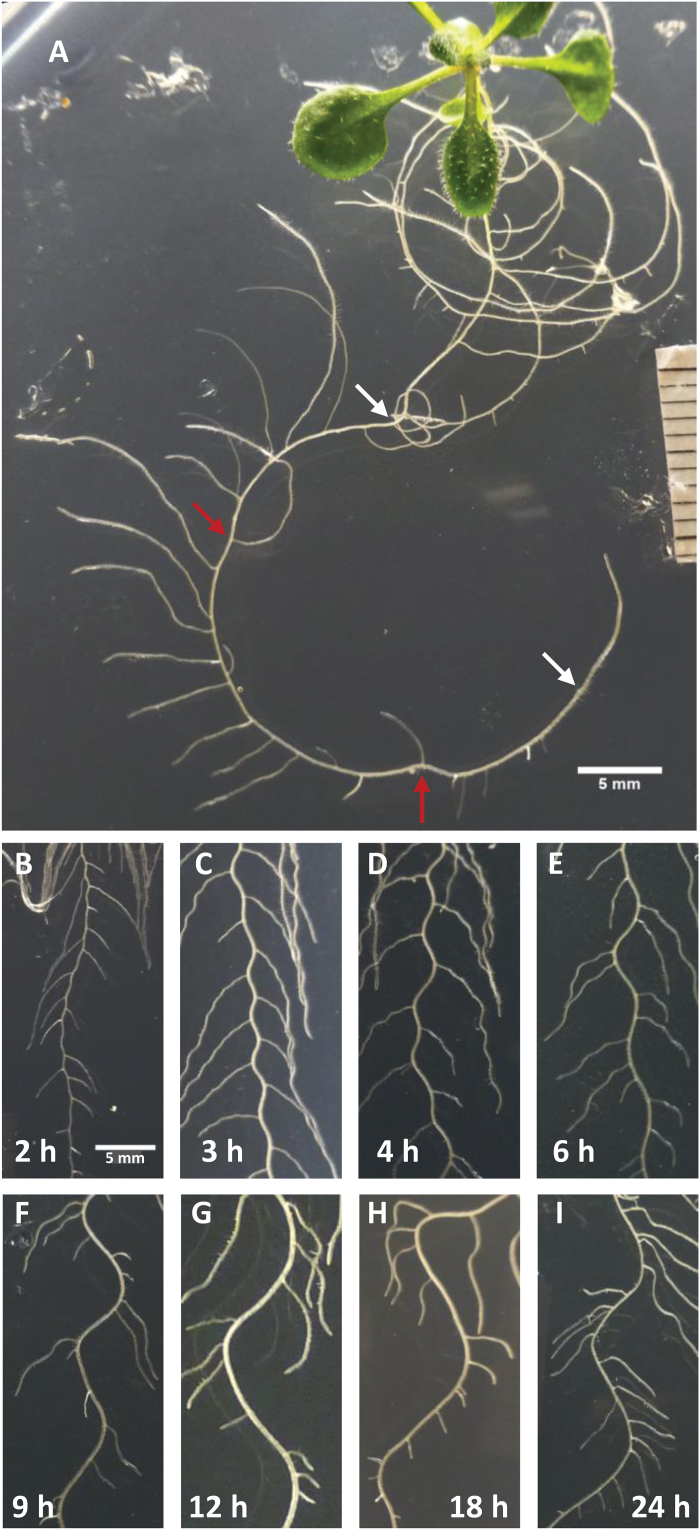
Modification of root architecture by experimental variation of gravitropic bending. (A) Circular growth of a root growing on a clinostat with a speed of 360° per 6 d during 5–10 daig (white arrows) and photographed after a further 2 d on the clinostat. Exceptionally inwardly orientated LRs at indentions are marked (red arrows). In addition, there are three degenerated LRs without obvious indentions. (B–I) Periodic gravitropic bending of roots rotated by 90° back and forth in the vertical orientation during 5–10 daig at indicated intervals.

**Fig. 4.**
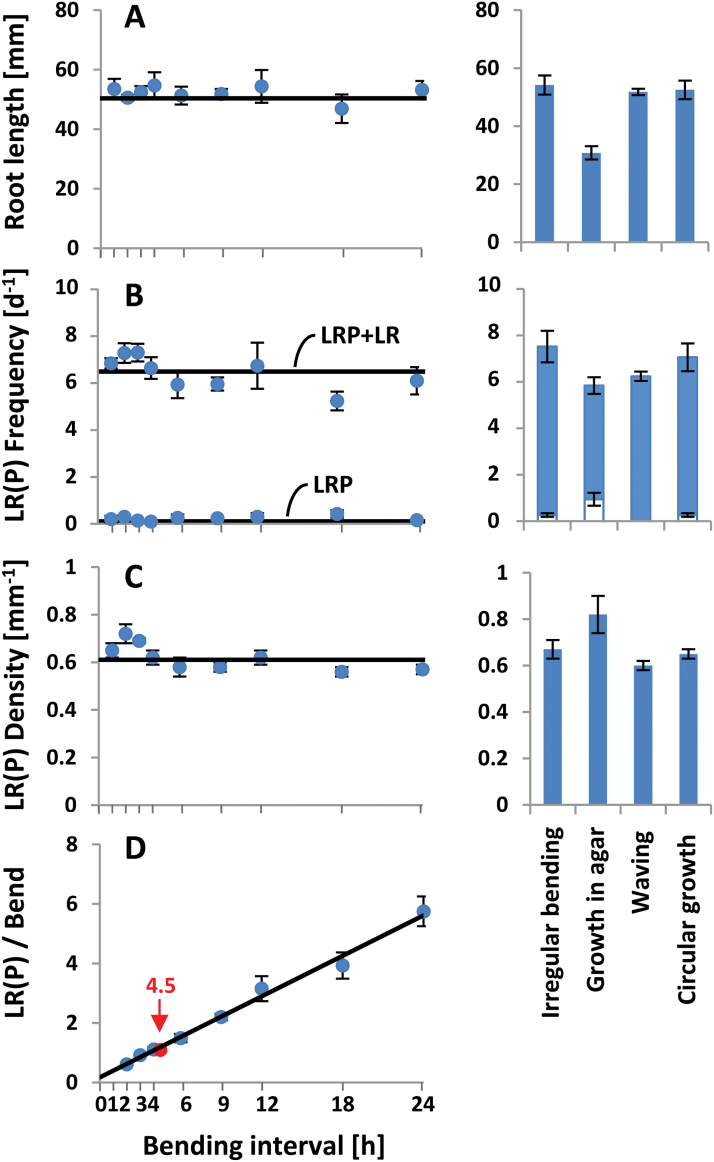
Effect of waving and gravitropically induced root bending illustrated in Figs 2A and 3B–I on primary root length (A), LR(P) frequency (B), LR(P) density (C), and the average number of LR(P)s per bend (D) between 5 and 10 daig, determined at 12 daig. The bending interval displayed in waving roots (Fig. 2A) is marked (4.5h, arrow). Values for other forms of bending shown in Figs 2 and 3 are depicted on the right (the fraction of LRPs is indicated by the white part of columns in B).

In the first experiment (shown in Fig. 3A), seedlings were grown on vertical plates (1% agar) on a clinostat rotating with a speed of 360° per 6 d without affecting the growth rate of the primary root (Fig. 4A, right). This causes continuous gravitropic readjustment of the growth direction and thus monotonic bending resulting in a circle, the radius of which depends on the speed of rotation (Fig. 3A). Under these conditions, i.e. in the absence of periodic root bending, virtually all LRs are produced at the outer (convex) side of the circle with the same frequency as observed in the waving or irregular meandering mode of growth (Fig. 4B, right). Notably, if LRs emerge exceptionally at the inner side, they are often located at indentions in the otherwise perfect circle. Related observations have been reported for coiling roots of the agravitropic *aux1* mutant (De Smet *et al.*, 2007; Ditengou *et al.*, 2008). This experiment shows that even very faint, monotonic bending is sufficient to restrict LRs to the convex root side whereby their average distance is not affected. In other words, bending directs LR initiation to the convex side, but does not influence the frequency of LR initiation.

In the second experiment, seedlings were similarly grown on a vertical turntable rotating the plate by 90° back and forth at defined intervals (Lucas *et al.*, 2008a). This forces the root to grow in a gravitropically induced periodic bending pattern, the frequency of which is dictated by the frequency of turning. The root responds to this treatment at intervals of between 2 and 24h (Fig. 3B–I) without changing its growth rate (Fig. 4A, right). The pattern of stationary spots of *pDR5* activity revealed by LUC expression coincided with the LR(P) pattern detectable at later stages (Supplementary Fig. S3), indicating that gravitropic bending did not affect the transformation of prebranch sites into LRs. In contrast to a previous report (Lucas *et al.*, 2008a), the frequency of LRs was the same at all bending frequencies, resulting in a constant LR density and a linear increase of the average number of LR(P)s per bend (Fig. 4B–D). The number of bends exceeded the number of LRs at 2-h intervals while often two LRs on each bend could be found at 6-h intervals. The relation of one LR(P) per bend was matched at an interval of 3.5±0.5h, and the relation determined for waving roots (1.09±0.03 LR(P)s per bend; Fig. 2A) fits into this curve at an interval of 4.5±0.3h. LR(P) frequency (6.5±0.3 d^−1^) and density (0.63±0.02mm^−1^ primary root length) were not significantly different in all experimental variations of root growth with the exception of growth within the agar medium, which caused a reduction of primary root length and LR(P) frequency, probably as a response to increased mechanical impedance (Fig. 4A, B, right). Since the unilateral thigmotropic effect is replaced here by an omnilateral effect, gravitropism dominates in orientating root growth, resulting in an attenuation of curving and an irregular LR(P) arrangement (Fig. 2B). Under these conditions, also the waving response and the strict alternating order of LR(P)s is suppressed, while the bending and ordered LR(P) arrangement enforced by periodic gravitropic stimulation is maintained (data not shown).

**Fig. 5.**
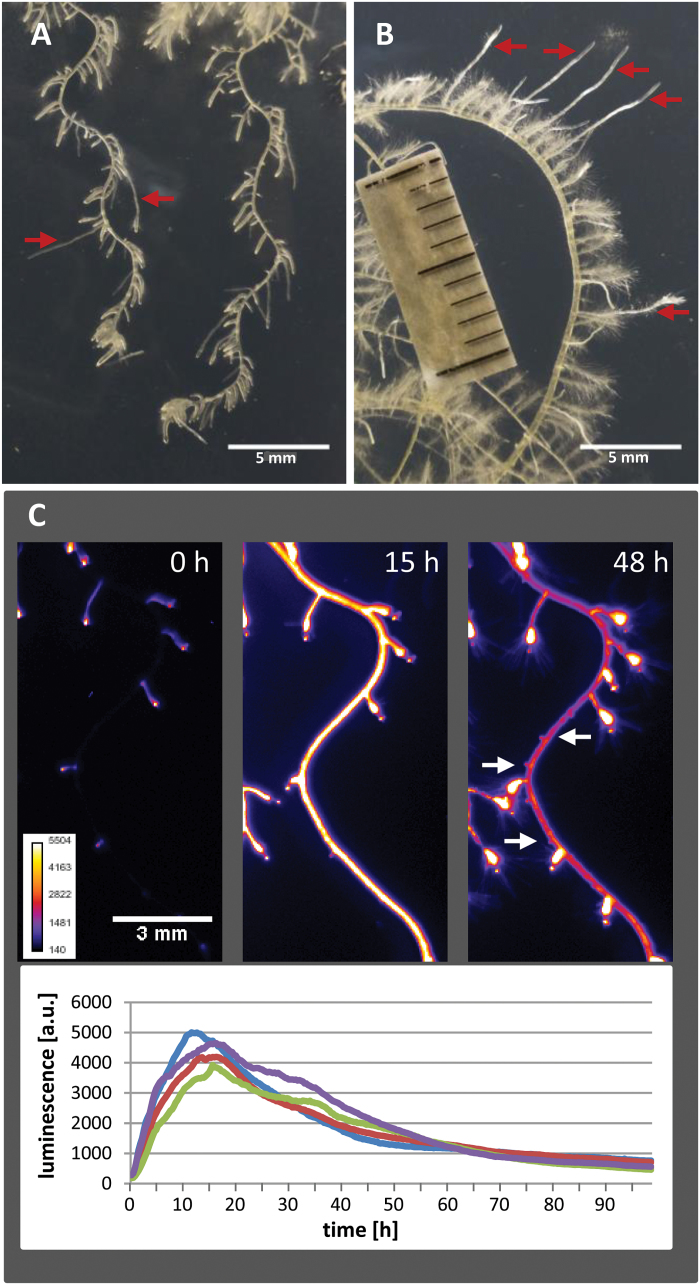
Positioning of newly formed LRs induced by auxin in the mature part of uniformly circular or alternating bending roots. Seedlings kept under circular bending conditions (A) or periodic gravitropic bending conditions (B) between 5 and 10 daig as shown in Fig. 3A and D, respectively, were overlayered on the plate with 10 µM auxin (1-naphthaleneacidic acid) for 10min. Pictures were taken after a further 3 d (A) or 4 d (B) of growth. Red arrows indicate LRs produced before auxin treatment. Panel (C) shows the effect of the auxin treatment on *pDR5:LUC* activation in the primary root of seedlings grown as in (B) before addition of auxin (left), or 15h (middle), and 48h (right) after the onset of auxin incubation. White arrows indicate newly formed primordia. The time course of LUC activity determined in four arbitrarily selected root regions without prebranch sites is shown below.

In the third experiment, seedlings grown under a monotonic circular bending or a periodic gravitropic bending regime as shown in Fig. 3A, G were briefly overlayered with an auxin solution that stops primary root growth and induces ectopic *de novo* formation of additional LRs from xylem pole pericycle cells in the mature region (Blakely and Evans, 1979; Laskowski *et al.*, 1995). Fig. 5A, B shows that the subsequently produced LRs are again positioned at the convex root flanks similar to the LRs previously produced in the growing root tip. Evidently, even high concentrations of auxin are unable to overcome this pre-established positional control. These experiments indicate that bending causes a long-lasting fixation of future LR sidedness, allowing separation in time of the processes involved in determining LR abundance from those involved in determining lateral LR positioning. While the frequency of LR initiation can be promoted by auxin, the positioning of LRPs appears to be under auxin-independent control. As shown in Fig. 5C auxin causes a uniform, transient *pDR5* activation in the entire primary root detectable less than 30min after the onset of treatment, culminating after 12–15h, and decreasing with a half-life of about 24h. This wave of auxin activity is obviously sufficient for unblocking LR initiation at sites predestined by the bending pattern.

**Fig. 6.**
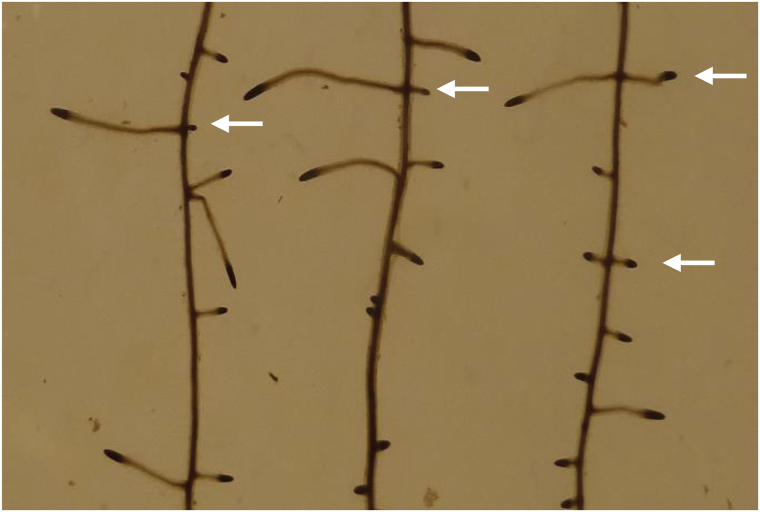
Formation of opposite LRs (arrows) on primary roots growing in the presence of the auxin transport inhibitor prenoylbenzoic acid (PBA). Five-day-old seedlings were transferred to agar medium containing 25nM PBA and photographed after a further 7 d (stained with acetocarmine). Similar results were obtained with naphthylphtalamic acid.

The bilateral priming reaction visualized by *pDR5* activation at opposite xylem poles (De Smet *et al.*, 2007) is followed by a unilateral LRP initiation that is always localized to the convex side of bends (Figs 2 and 3). Obviously, the convex and the concave flank of bends differ with respect to their ability to support LRP initiation. This can best be explained by assuming that the decision between convex and concave initiation is realized by inhibiting founder cell initiation at the concave side rather than promoting it at the convex side. This interpretation is supported by an unexpected observation. Seedlings growing on media containing auxin transport inhibitors or high auxin concentrations often produce opposite pairs of LRs, demonstrating various degrees of retardation on one side (Fig. 6). This indicates that the inhibition of LR formation on one side can be partly or totally abolished under certain experimental conditions. Interestingly, the occurrence of opposite twin primordia has previously been observed in a mutant lacking the receptor-like kinase ACR4, which is involved in restricting formative cell divisions to the sites of LRP initiation (De Smet *et al.*, 2008).

Taken together, these results provide evidence that both the determination of LR frequency by periodic LR priming and LR positioning by bending are involved in shaping the root system and that these factors can be experimentally discriminated on the basis of their distinct functions in pattern formation.

## Discussion

The exhibition of waving root growth has been interpreted in various ways (Oliva and Dunand, 2007), including the implication of an endogenous clock (Moreno-Risueno *et al.*, 2010). In agreement with most published experimental material, waving growth can be explained as a complex interaction between gravitropic and thigmotropic responses at the surface of an elastic solid surface (Thompson and Holbrook, 2004). Oscillatory behaviour of biological systems is generally caused by the interplay of an inducing reaction (I), connected to a counteracting reaction (II) forming a feedback loop in which a delay between I and II prevents settling on a steady state (Novak and Tyson, 2008). Similar to the well-known obstacle-avoidance reaction (Massa and Gilroy, 2003), the root tip pushed along an inclined, impenetrable agar surface experiences gravitropically enforced friction at the side touching the medium and the elongation zone responds to this tactile force by bowing sideward and thereby slipping the root tip to the opposite side (I). This change in growth direction minimizes friction and activates overshooting gravitropic curving back to the vertical orientation (II), reinitiating friction and an avoidance reaction towards the opposite side. At constant growth rate this sequence of alternating, transient changes in growth direction produces a regular oscillatory left–right pattern of bends in the plane of the agar surface. Exemplifying the principles of biological oscillations, waving root growth represents a laboratory artifact produced only under specifically selected experimental conditions.

Although the bending hypothesis is seemingly sufficient for explaining LR pattern formation under waving conditions, it is clearly insufficient for covering situations where the primary root grows without, or with deviating, bending periodicity (Figs 2–4). By experimentally modifying the geometric path of root growth, it could be shown that the determination of LR frequency can be uncoupled from the exact positioning of LRs along the primary root, indicating independent regulation of these processes. The frequency of LR formation was constant at very different growth modi, providing strong evidence that periodic priming events are setting the overall pace of LR formation as proposed by the oscillation hypothesis. Using the *pDR5:LUC* reporter line, it has been reported that the priming oscillator in the root tip operates with a cycling time of ~6h, in agreement with the pace of pre-branch site formation (Moreno-Risueno *et al.*, 2010). In the experiments with genetically unmodified Columbia seedlings grown in continuous bright light, the average time interval between LR(P)s was 3.7h, suggesting that the cycling time of the supposed oscillator mechanism can be modified by the experimental conditions.

Independently of periodic priming, the trajectory of primary root growth does exert an important influence on pattern formation by determining the exact placement of LRs along the primary root. Firstly, bending determines the lateral positioning of LRs, presumably by preventing their realization at the concave side of bends. As illustrated in Fig. 3A, even very faint bending that can easily be overlooked in apparently straight growing roots can elicit this effect. Secondly, bending has a fine-tuning effect on longitudinal LR positioning by directing LRP initiation towards the top of bends. A comparison between waving and irregularly bending roots (Fig. 2A and D, respectively) reveals that the waving growth rhythm significantly contributes to the rhythmic, equidistant spacing of LRs along the primary root. As already noted by Noll (1900) (Fig. S4), this effect can lead to a clustering of LRs with a strong bias for the top of bends if the number of LRs exceeds the number of bends (Figs 3E–I and 5B). This clustering effect may be responsible for the finding that manual bending in a region of 1–5mm behind the root tip is followed by an increased appearance of convex LRs in this region (Ditengou *et al.*, 2008; Laskowski *et al.*, 2008). An increase in total number of LRs, indicative of an increase in the frequency of LR formation, has not been shown in these experiments. In agreement with Moreno-Risueno *et al.* (2010), we could not find any indication that bending induces *de novo* production of LRs (Table 1). Root elongation by 5mm takes about 10h while new LRPs are initiated every 3.7h (Fig. 1B). Hence, up to three priming events can take place, and founder cells can then be initiated and appropriately positioned, during the time needed to produce the root region involved in the bend. In summary, these observations necessitate the assumption that the priming oscillator predetermines in the longitudinal spacing of LRP initiation sites by establishing a supracellular field of competence in the rhizogenic pericycle cell lines within which mechanical cues can define the exact position of LR initiation. This could involve the receptor-like kinase ACR4, which is required for preventing formative cell division in the surroundings of induced founder cells (De Smet *et al.*, 2008).

The molecular mechanisms involved in pre-conditioning of particular root regions to LRP initiation and the spatial distribution of initiation sites in the pericycle are presently understood only in fragmentary terms (reviewed by Van Norman *et al.*, 2013). A recent report provides evidence that auxin is needed for translating the priming events into a corresponding sequence of pre-branch sites (Xuan *et al.*, 2015). It has long been known that auxin plays a central role as a mediator of LR development, although the precise point(s) of auxin action and the role of auxin transport to sites of auxin action have not yet been sufficiently clarified (Lavenus *et al.*, 2013). Based on the finding that the convex flanks of curved root regions are preferred sites of LR development, Richter *et al.* (2009) suggested an overview model of dual causation of LR patterning by (i) an endogenous developmental programme with the ability to respond to (ii) environmental cues such as mechanical forces mediated by Ca^2+^ signalling. However the interconnections of the signal cascades underlying these processes and the possible function of auxin as a connecting link remained a matter of conjecture. The *pDR5* activation in the oscillation zone is now believed to represent the output of the endogenous programme that does not involve oscillating peaks in auxin and can be observed prior to growth re-orientation during root waving (Van Norman *et al.*, 2013). This implies that auxin does not control endogenous pre-patterning, but is restricted to act in later stages of LR development including bending-related processes. On the other hand, mutants with defects in auxin transport or signalling still show wild-type bending-related LR formation suggesting that auxin-dependent signalling is not required for placing LRs at bends (Ditengou *et al.*, 2008; Richter *et al.*, 2009). Evidently, the close temporal and spatial coincidence of priming and bending in the elongation/oscillation zone of the growing root greatly hampers the functional separation of these events. Our results contribute to this discussion by characterizing an experimental system in which auxin is necessary and sufficient for inducing a rapid, priming-like *pDR5* activation followed by ectopic LR formation in the non-growing root section on the background of an existing spatial pre-pattern that determines the positioning of these LRs. Clearly, this system differs from the growing root tip by exhibiting uniform, extended *pDR5* activation, requiring relatively high auxin concentration and affecting pericycle cells presumably arrested in the G2 phase of the cell cycle (Blakely and Evans, 1979). Bearing these differences in mind, this system provides evidence that auxin can redirect pericycle cell development towards LR initiation in the mechanically static root. Taken together, our results emphasize that periodic priming and bending-dependent LR positioning co-operate in LR patterning by responding to separate input signals transmitted by separate signalling pathways.

The mechanism by which mechanical cell deformations can produce site-specific changes in cell development are presently unknown. Richter *et al.* (2009) suggested that increases in cell tension could elicit signalling by changes in cytosolic Ca^2+^ levels. However, it appears difficult to imagine how these transient changes could lead to a stable fixation of the positioning pre-pattern. An alternative, plausible mechanism could implicate the cortical microtubule cytoskeleton in translating mechanical changes produced by bending into signals controlling founder cell initiation. In growing axial plant organs such as roots, bending induced by gravity or physical forces causes rapid and lasting reorientation of microtubules from the transverse to the longitudinal direction specifically at the concave side of the organ (Fischer and Schopfer, 1997). A longitudinal orientation of the cortical microtubules could interfere with the anticline (perpendicular to the long axis) plane of cell divisions involved in founder cell initiation. This idea deserves experimental testing.

## Supplementary data

Supplementary data are available at *JXB* online.

Figure S1. Staining of LRPs with acetocarmine 20mm behind the root tip (above) and close to the root tip (below).

Figure S2. Detection of prebranch sites and LRPs by pDR5:LUC imaging and acetocarmine staining, respectively.

Figure S3. Generation of prebranch sites and their conversion into LRs in a root subjected to periodic gravistimulation (4-h intervals, Fig. 3D).

Figure S4. Pattern of LR formation in soil-grown seedlings of *Lupinus albus* demonstrating the clustering response at the convex side of gravitropically induced bends.

Video S1. Development of the root system of an Arabidopsis seedling.

Supplementary Data

## Abbreviations:

daig: days after initiation of germination
LR: lateral root
LRP: lateral root primordium.
