# Supplementary material for: Priming and positioning of lateral roots in Arabidopsis. An approach for an integrating concept
Source: J Exp Bot. 2015 Dec 27;67(5):1411–20. doi: 10.1093/jxb/erv541 (PMC4762386; doi:10.1093/jxb/erv541)
Supplement: Supplementary Data [file supp_67_5_1411__index.html]

Priming and positioning of lateral roots in Arabidopsis. An approach for an integrating concept — Priming and positioning of lateral roots in Arabidopsis. An approach for an integrating concept — Supplementary Data 

# Priming and positioning of lateral roots in Arabidopsis. An approach for an integrating concept

## Supplementary Data

Data files

- Supplementary\_figures\_S1\_S4.pdf - Supplementary Data
- supplementary\_video\_S1.avi - Supplementary Data
