# Supplementary material for: Priming and positioning of lateral roots in Arabidopsis. An approach for an integrating concept
Source: J Exp Bot. 2015 Dec 27;67(5):1411–20. doi: 10.1093/jxb/erv541 (PMC4762386; doi:10.1093/jxb/erv541)
Supplement: Supplementary Data [file supp_erv541_Supplementary_figures_S1_S4.pdf]

Journal of Experimental Botany

Manuscript title and authors:

Priming and positioning of lateral roots in Arabidopsis. An approach for an integrating concept.

Stefan Kircher and Peter Schopfer

Supplementary Data

Fig. S1

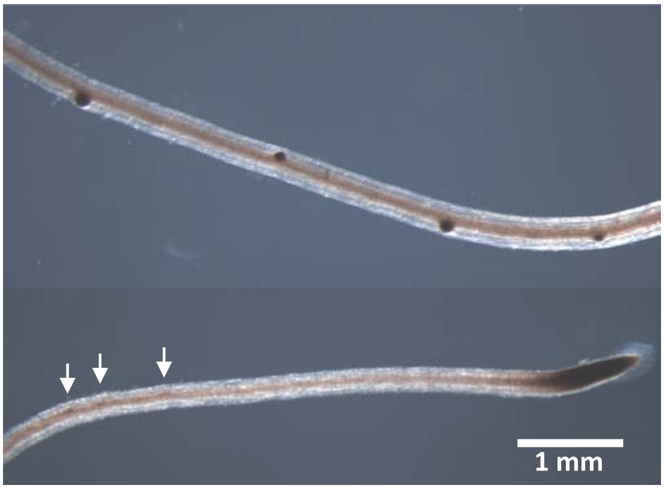

Fig. S1. Staining of LRPs with acetocarmine 20 mm behind the root tip (above) and close to the root tip (below).

Fig. S2

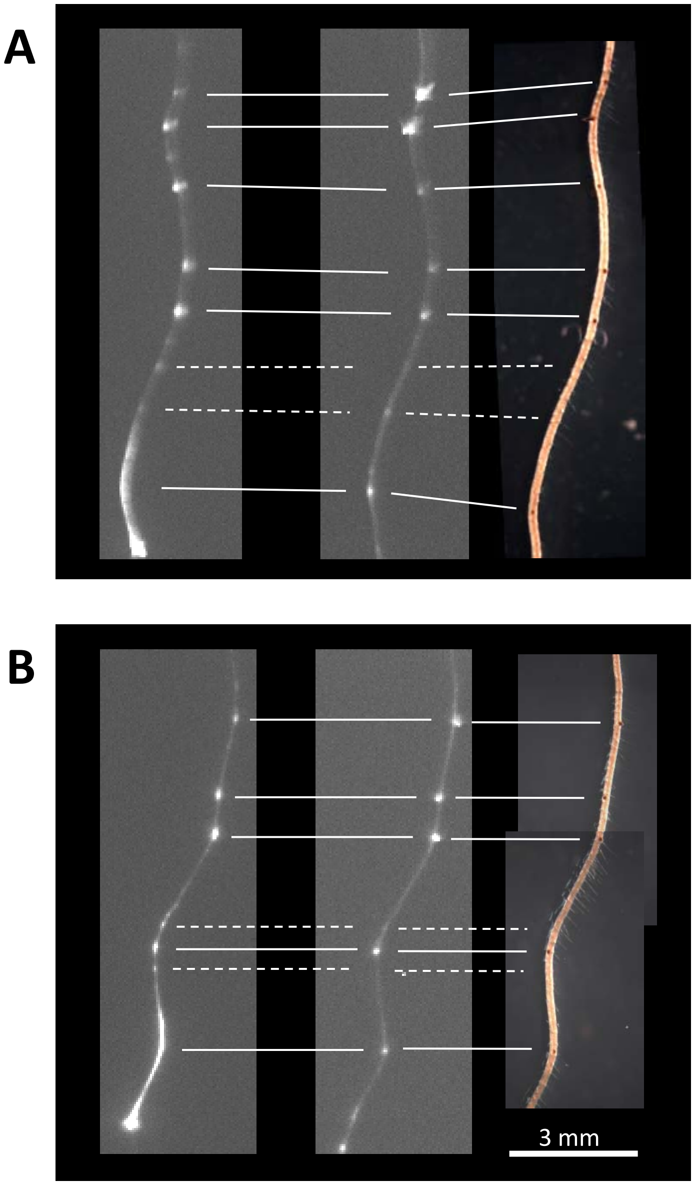

Fig. S2. Detection of prebranch sites and LRPs by *pDR5:LUC* imaging and acetocarmine staining, respectively. In the two examples (*A*,*B*) a segment close to the root tip of a 5-d-old seedling harboring the *pDR5:LUC* transgene was analyzed for LUC activity immediately (*left*) and after 24 h of additional growth (*middle*) followed by fixation and staining (*right*). The distance between the latest prebranch site and the first LRP detectable by staining is in the range of 3-4 mm. The dashed lines mark non-persistent spots of LUC activity that did not produce LRPs.

Fig S3

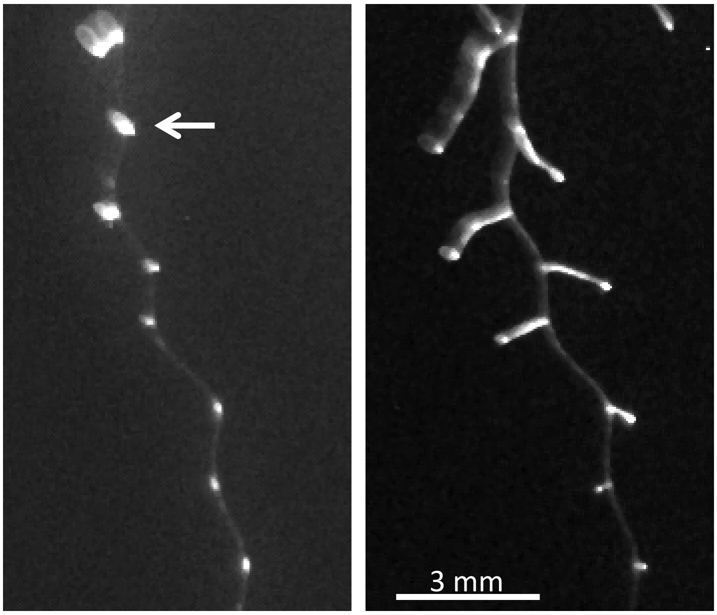

Fig. S3. Generation of prebranch sites and their conversion into LRs in a root subjected to periodic gravistimulation (4-h-intervals, Fig. 3*D*). *pDR5:LUC* expression was imaged after 24 h of gravistimulation (*left*, start at arrow) and again 24 h later (*right*).

Fig. S4

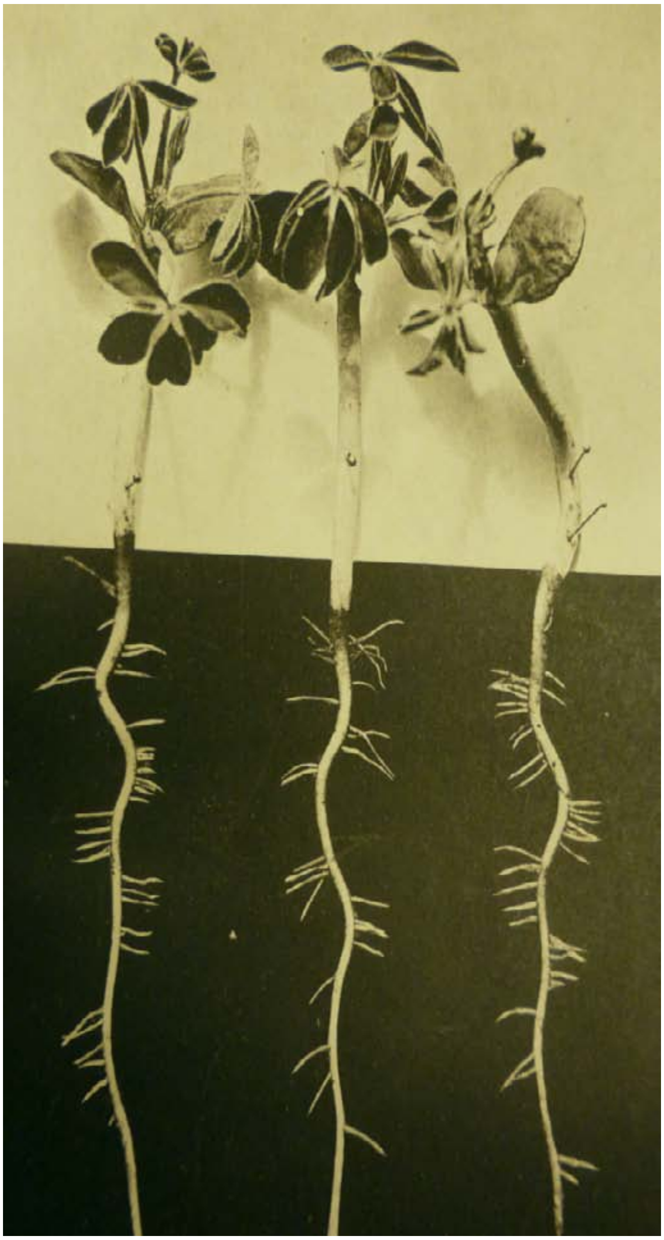

Fig. S4. Pattern of LR formation in soil-grown seedlings of *Lupinus albus* demonstrating the clustering response at the convex side of gravitropically induced bends. Picture reproduced from a publication by F. Noll (1900) describing also many other basic features of LR generation rediscovered in recent years in Arabidopsis.
